# Uterine Müllerianosis

**DOI:** 10.3390/jcm15176918

**Published:** 2026-09-07

**Authors:** Ivo Vukasović, Držislav Kalafatić, Vladimir Banović

**Affiliations:** 1 Department of Obstetrics and Gynecology, University Hospital Centre Zagreb, 10000 Zagreb, Croatia; ivo.vukasovic@kbc-zagreb.hr (I.V.);; 2 School of Medicine, University of Zagreb, 10000 Zagreb, Croatia

**Keywords:** müllerianosis, endometriosis, adenomyosis, uterine diseases, histopathology, ultrasonography

## Abstract

**Introduction**: Müllerianosis is a rare pathological entity characterized by ectopic Müllerian-derived tissues, including endometrium, endocervix, and/or endosalpinx. Most reported cases involve the urinary bladder, while uterine involvement is extremely uncommon. We present a case of müllerianosis presenting as a cystic lesion of the anterior uterine wall, and we provide a review of the available literature. **Case Presentation**: We report a 37-year-old woman with an asymptomatic anterior uterine wall lesion detected 11 years ago on routine ultrasound. Initially measuring 60 × 35 mm, the lesion was surgically excised and histopathologically confirmed as müllerianosis, containing endometrial and tubal-type tissue. Eleven years later, she presented with abnormal uterine bleeding. Ultrasound revealed a heterogeneous lesion with cystic areas and uterine asymmetry suggestive of adenomyosis. Conservative management, including curettage, failed, necessitating total abdominal hysterectomy. Histopathology confirmed adenomyosis and endometriosis in addition to prior findings. **Discussion**: This case is notable for the long follow-up, the uncommon anterior uterine location, and the large lesion size. It supports an embryologic or metaplastic origin of müllerianosis and suggests potential links with other uterine pathologies. Surgical excision remains the treatment of choice, while imaging, histopathology, and immunohistochemistry are essential for accurate diagnosis. Long-term monitoring is recommended due to recurrence risk and the rare potential for malignant transformation. **Conclusions**: Our report emphasizes the importance of awareness, careful diagnosis, and extended follow-up in managing rare uterine Müllerian lesions.

## 1. Introduction

Müllerianosis is a rare pathological entity defined by the presence of distinct Müllerian-derived tissues located ectopically, outside their normal anatomical locations. It was first characterized by Clement and Young in 1996 [1]. Histologically, the condition comprises endometrial, endocervical, and/or endosalpingeal components. Fewer than several dozen cases have been documented in the literature, most frequently involving the lower urinary tract [2,3]. Because of its variable clinical presentation and its potential to mimic malignant neoplasms, müllerianosis represents a significant diagnostic challenge [4]. The differential diagnosis of an atypical uterine mass is clinically relevant because benign and malignant myometrial lesions may share overlapping clinical and sonographic features. Uterine sarcomas may appear as heterogeneous masses with cystic areas and variable vascularity, potentially mimicking benign or atypical lesions [5]. Metastatic disease may also present as an unusual uterine mass; Ludovisi et al. described breast cancer metastasis to a uterine leiomyoma with sonographic features resembling a primary smooth-muscle tumor [6]. Recent clinical-ultrasound algorithms further emphasize the role of integrated clinical and sonographic assessment in identifying uterine sarcomas and STUMP [7]. Müllerianosis is particularly challenging because its uncommon location and variable appearance may mimic other uterine lesions. Histopathology, with immunohistochemistry when necessary, remains essential for definitive diagnosis and appropriate management. We present a rare case of uterine müllerianosis manifesting as a cystic intramyometrial lesion of the anterior uterine wall, one of the rarest reported anatomical locations, with an exceptional 11-year longitudinal follow-up. We also review the available literature to summarize the current understanding of this uncommon disorder.

## 2. Case Report

We report the case of a 37-year-old female patient (BMI 29.3 kg/m^2^) who was admitted to our clinic via the emergency department in September 2025 for severe and irregular uterine bleeding, ongoing for the past 20 days. The patient reported using more than 10 sanitary pads per day in recent days. Despite having started treatment with medroxyprogesterone acetate approximately 10 days earlier, the bleeding had only partially decreased in intensity. Laboratory investigations demonstrated secondary anemia with a hemoglobin level of 87 g/L.

The patient was otherwise healthy, with no significant past medical history. In September 2014, a routine transvaginal ultrasound revealed an asymptomatic solid–cystic mass measuring 60 × 35 mm with prominent vascularization on the anterior uterine wall. A laparotomy with resection of the mass was performed. Histopathological analysis revealed a fibrous capsule composed of granulation tissue, containing focal glandular structures lined by cylindrical epithelium of endometrial and tubal type. Immunohistochemically, the stromal component was positive for CD10, SMA, and desmin, while CD117 and HMB-45 were negative. Ki-67 demonstrated proliferative activity within the granulation tissue layer of the capsule. Following the surgical procedure, the patient had three deliveries, each by cesarean section. During the last cesarean, Pomeroy sterilization was performed. In the past several months prior to admission, she developed irregular uterine bleeding and underwent curettage in July 2025, which revealed normal histopathology, including fragments of late-proliferative-phase endometrium.

Upon current admission, a transvaginal ultrasound examination was performed by a gynecologist with 20 years of experience in gynecological ultrasonography. The examination was performed using a Voluson Expert 21, and representative gray-scale and color Doppler images were digitally stored for subsequent review. The uterus was globularly enlarged, with asymmetrical walls (anterior wall 65 mm, posterior wall 17 mm), with a total volume of 476 cm^3^. A heterogeneous, echogenic lesion with cystic areas measuring 75 × 84 × 69 mm was noted in the anterior wall, with a regular posterior acoustic shadow. Color Doppler examination demonstrated abundant vascularity (color score 4), with both circumferential and intralesional vascularity present. The endometrium measured 5 mm with an interrupted endometrial–myometrial interface. The uterotomy scar appeared normal, and both adnexa were unremarkable. Representative gray-scale and color Doppler images and cine clips were digitally stored and subsequently reviewed for analysis. Given the lesion’s large size, heterogeneous appearance, cystic areas, and abundant vascularity, the preoperative differential diagnosis included both a benign atypical uterine lesion and a malignant uterine mesenchymal tumor. Beta-hCG on admission was negative, while serum CA-125 was not part of the initial laboratory assessment. An urgent curettage was performed; however, the patient continued to experience bleeding. An intrauterine Foley catheter was placed for 24 h to control further hemorrhage. Histopathological examination revealed endometrium with signs of secretory activity and focal metaplastic changes of the epithelium. Due to a further decline in hemoglobin levels, the patient received red blood cell transfusions on two occasions.

Given the failure of medical therapy and curettage, ongoing hemorrhage, and development of severe anemia, a laparotomy with total abdominal hysterectomy was performed. Intraoperatively, the uterus was enlarged, with serosal changes suggestive of endometriotic foci, and the vesicouterine fold adhered densely to the anterior uterine wall. The procedure and postoperative course were uneventful. At a follow-up visit two months after surgery, the patient was reported to be in excellent condition. The final pathological finding describes a whitish submucosal oval mass measuring 8.8 cm in diameter that occupies the entire uterine cavity and is composed of intersecting bundles of smooth muscle cells with occasional islands of well-formed endometrial glands and stroma, with the overlying endometrium thinned and exhibiting a basal-type appearance, consistent with a diagnosis of uterine adenomyoma.

### Literature Review

A structured narrative literature review was conducted to identify published cases and studies concerning müllerianosis, with particular emphasis on uterine involvement, clinical presentation, imaging findings, histopathological characteristics, differential diagnosis, treatment, and follow-up. The literature search was performed using PubMed/MEDLINE, Scopus, and Google Scholar and included publications from 1990 to 2025. The search strategy included the term “müllerianosis” and related terms describing Müllerian lesions of the uterus. A total of 90 publications were identified. After screening for relevance according to the predefined inclusion criteria, 63 publications describing 69 individual cases were considered relevant and included in the final narrative review. Publications that were not directly relevant to müllerianosis or did not provide sufficient clinical, imaging, histopathological, treatment, or follow-up information were excluded. The identified cases were used for descriptive purposes only and were not considered a representative epidemiological cohort. Given the rarity of müllerianosis and the predominance of case reports and small case series, the possibility of selection and publication bias was acknowledged.

## 3. Discussion

### 3.1. Etiology and Pathogenesis

The etiopathogenesis of müllerianosis has intrigued clinicians since its earliest descriptions. Multiple hypotheses have been proposed, reflecting the complex and multifactorial nature of the disease (Table 1). In 1996, Young and Clement defined müllerianosis as the presence of at least two of the three Müllerian tissues—endometrium, endosalpinx, and endocervix and suggested that the condition may result from the implantation of Müllerian tissue, potentially following surgical procedures [1]. Batt et al. later refined this concept by characterizing müllerianosis as an organoid structure of embryonic origin, a choristoma composed of Müllerian rests (endometrium, endosalpinx, and/or endocervix), occurring either singly or in combination—thereby emphasizing a developmental rather than an acquired etiology [3]. Donne et al. introduced an alternative metaplastic hypothesis, suggesting that Müllerian metaplasia may give rise to tubal, endometrial, or endocervical tissues. The predilection of lesions for the posterior bladder wall and fundus corresponds to areas of peritoneal coverage known to be particularly responsive to female sex hormones [8].

Scott et al. proposed an embryological origin of müllerianosis, suggesting that aberrant activation of developmental signaling pathways (including WNT and HOX genes) during organogenesis may result in ectopic Müllerian tissue [9]. Similarly, Barresi et al. argued that displacement of coelomic epithelium and its associated mesenchyme during embryogenesis could explain Müllerian tissue developing at ectopic sites, including the spinal cord [10]. Evidence supporting a developmental origin of Müllerian lesions has also been provided by studies demonstrating misplaced endometrial tissue in human female fetuses. Signorile et al. identified ectopic endometrial tissue in several pelvic locations in female fetuses, including the uterine wall, supporting the hypothesis that endometrial tissue may become displaced during organogenesis [11]. Similarly, Bouquet de Jolinière et al. demonstrated displaced endometrial glands and embryonic duct remnants in the female fetal reproductive tract, further supporting a possible embryological contribution to the development of endometriotic and Müllerian lesions [12].

An interesting feature of our case is that the tumor-like lesion developed before the patient underwent any uterine surgical procedures, including three subsequent cesarean sections and the later laparotomy during which the mass was excised. This temporal sequence strongly supports an embryological or metaplastic origin rather than implantation as the underlying etiopathogenic mechanism. An iatrogenic implantation mechanism, analogous to that proposed for parasitic myomas following tissue disruption during previous surgery, is less likely in our patient because the Müllerian lesion was identified before any uterine surgical intervention [13].

**Table 1:** Summary of the principal etiopathogenetic theories proposed for Müllerianosis.

Implantation Theory	Metaplastic Theory	Embryologic Theory
Müllerian epithelium is transported from the genital tract and implanted into ectopic locations.	Local mesothelium or submesenchymal tissue differentiates into Müllerian-type epithelia (endometrium, endosalpinx, endocervix).	Müllerian tissues are misplaced during organogenesis and incorporated into other organs.
- prior pelvic surgery (e.g., cesarean section) or endometriosis	- presence of multiple Müllerian tissues in one lesion (endometrium, tubal, endocervical) - typical location in posterior bladder wall and dome, hormonally responsive area	- ectopic endometrium found in human fetuses outside uterine cavity - explains bladder or distant site lesions in absence of surgery - distinct from endometriosis; not due to retrograde shedding but embryologic misplacement
▪ Young and Clement [1] ▪ Jimenez-Heffernan et al. [14] ▪ Islam et al. [15]	▪ Donné et al. [8] ▪ Nogales et al. [16] ▪ Koren et al. [17]	▪ Barresi et al. [10] ▪ Batt et al. [3] ▪ Scott et al. [9]

### 3.2. Clinical Presentation

In the analyzed cohort of 69 müllerianosis cases (Supplementary Materials), patient age at diagnosis ranged from 11 to 84 years, with a mean of 43.5 years, indicating a predominance in reproductive and perimenopausal age but without strict age limitation. Approximately 40.6% of patients had a history of pelvic or abdominal surgery, most commonly cesarean section. Although this suggests a potential association between prior surgery and the development of müllerianosis, the relationship remains inconclusive given the considerable number of patients without any surgical history.

Analysis of the reported cases indicates that urinary manifestations constitute the predominant clinical presentation, most commonly dysuria, hematuria, urinary urgency, and pain [2]. Notably, in a substantial proportion of patients, symptoms exhibit a catamenial pattern, occurring exclusively or predominantly during menstruation, thereby supporting the hypothesis of hormonal responsiveness of the ectopic Müllerian tissue (e.g., cyclical hematuria or cyclical dysuria) [18,19,20,21,22,23,24,25,26]. The symptomatology further varies according to lesion location, with gastrointestinal manifestations (such as defecatory difficulty and rectal bleeding) and, more rarely, neurological symptoms, including cyclical neurological deficits, documented in select cases [9,10,27]. A number of cases were entirely asymptomatic, with lesions identified incidentally during evaluation for unrelated conditions.

The most common site of müllerianosis was the urinary bladder, accounting for more than half of all reported cases. Within the bladder, the posterior wall represented the predominant single location (Supplementary Materials). Other intravesical sites included the lateral wall, dome, and trigone. Extrapelvic or extravesical locations were considerably less frequent, with reported lesions in the ureter, uterus, adnexa, appendix, and lymph nodes, as well as exceptionally rare sites such as the liver, lung, retroperitoneum, and spinal cord [4,9,10,28,29,30,31,32,33,34].

In our case report, an asymptomatic lesion of the anterior uterine wall was detected during a routine ultrasound examination. To our knowledge, only three other cases of uterine müllerianosis have been reported to date: the first involved a cystic lesion of the posterior uterine wall (cystic endosalpingiosis) in a patient with pelvic pain; the second was an asymptomatic cystic endosalpingiosis of the uterine fundus discovered incidentally during pregnancy; and the third presented as a multiloculated cystic mass on the serosal surface of the uterine fundus containing both endometrial and tubal-type epithelium in a patient with pelvic pain [27,35,36]. However, our case is exceptional because the lesion was located entirely within the myometrium of the anterior uterine wall, whereas the previously reported uterine Müllerian lesions were confined to the uterine surface, cervix, or uterine parametrium rather than the myometrium itself. Interestingly, the largest reported lesions have been those involving uterine müllerianosis, which is consistent with our case, in which the lesion measured 6 cm.

### 3.3. Diagnostic Evaluation

Across the collected cases, imaging findings demonstrated substantial heterogeneity, reflecting the diverse anatomical locations and morphological patterns of müllerianosis. Ultrasound was the most frequently used initial modality, typically revealing echogenic, cystic, or polypoid masses, most often arising from the posterior or posterolateral bladder wall or the bladder dome (Supplementary Materials). CT and MRI most frequently described soft-tissue or mixed solid–cystic masses involving the bladder, ureter, or adjacent pelvic structures, often with features of heterogeneous enhancement or glandular/cystic morphology. These modalities also helped delineate local extension—such as involvement of the parametrium, abdominal wall, or adnexa—and occasionally identified ureteral obstruction or filling defects suggestive of secondary involvement. Imaging most often demonstrates features that frequently mimic neoplastic pathology (Supplementary Materials). Cystoscopy, when performed, commonly confirmed polypoid, nodular, mucinous, or cystic intravesical lesions, often consistent with submucosal or posterior wall involvement (Supplementary Materials).

**Figure 1:**
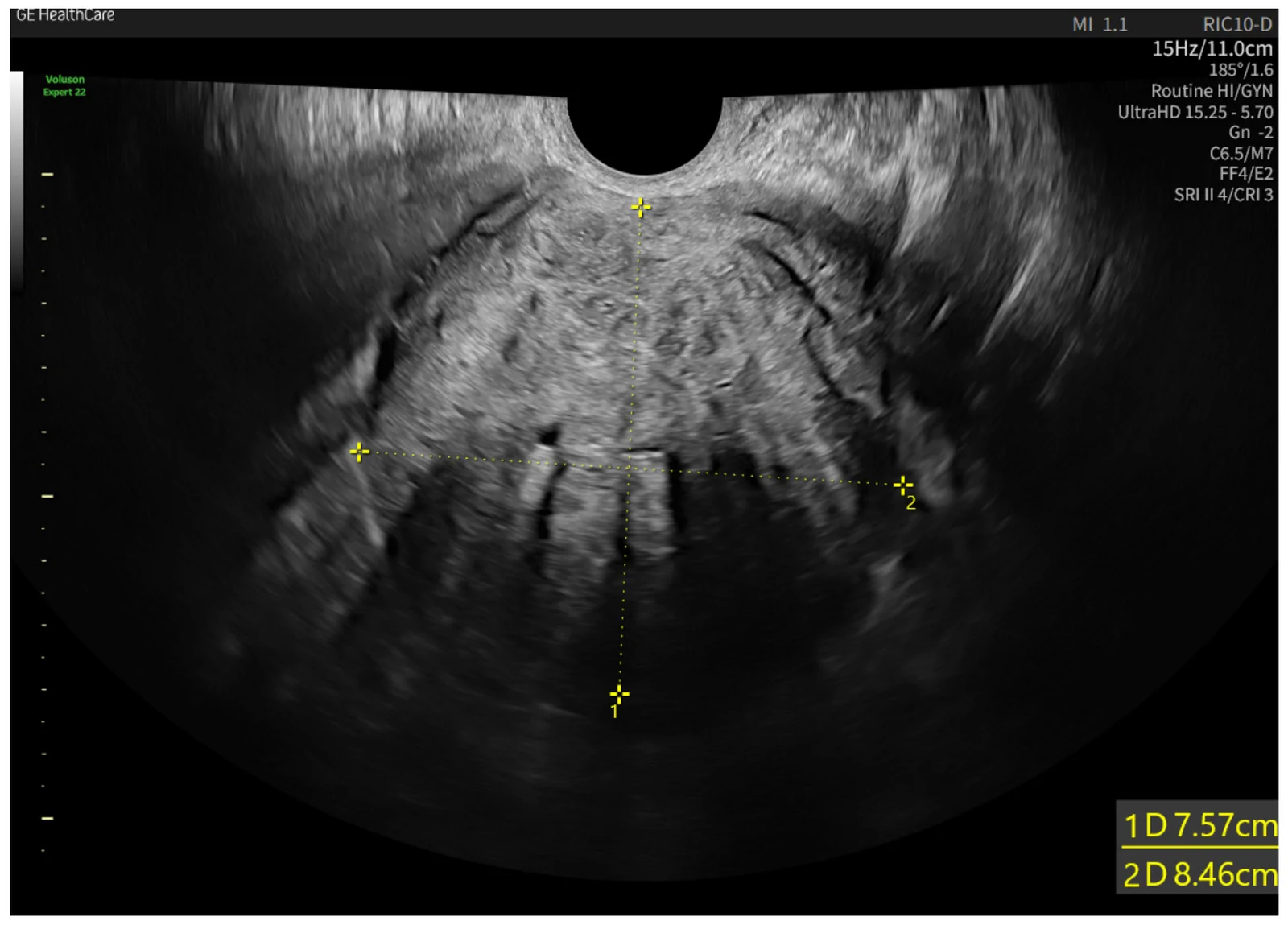
Transvaginal ultrasound appearance of the anterior uterine wall lesion.

In our case, ultrasound was the initial imaging modality that detected uterine pathology, first 11 years ago with an unusual-appearing mass on the anterior uterine wall measuring 60 × 35 mm, and again recently, when it revealed asymmetry between the anterior and posterior uterine walls with sonomorphologic features suggestive of adenomyosis (Figure 1). Given the patient’s history of müllerianosis and the location of the first lesion, we interpreted the later ultrasound findings with caution, despite the presence of direct and indirect signs of adenomyosis according to the MUSA criteria [37]. The sonographic differential diagnosis of an atypical uterine mass may be particularly challenging because some uterine sarcomas can present as heterogeneous solid lesions with cystic areas and increased vascularity, overlapping with the appearance of benign or degenerative uterine masses [5]. In our case, the heterogeneous echogenic appearance, cystic areas, marked vascularity, and progressive enlargement of the lesion raised concern for an atypical uterine mass, and a malignant mesenchymal tumor could not be excluded preoperatively. However, ultrasound findings alone cannot reliably distinguish müllerianosis or other benign uterine lesions from sarcoma. Therefore, careful clinical and sonographic assessment should be considered a risk-stratification tool rather than a definitive diagnostic method, with histopathological examination remaining essential for final diagnosis. Although heterogeneous and nonspecific, ultrasound remains an accessible and cost-effective method for detecting lesions (whether müllerianosis or adenomyosis) and for long-term monitoring of uterine structural changes.

Emerging approaches based on machine learning and radiomics may further improve the preoperative characterization of uterine masses. Machine-learning models have shown potential for differentiating uterine leiomyomas from sarcomas, while radiomic analysis has also demonstrated potential in predicting treatment response and identifying clinically relevant subgroups in gynecologic malignancies [38]. Although such approaches are not currently applicable to the diagnosis of müllerianosis, they may represent a promising direction for future research in the differential diagnosis of rare and atypical uterine lesions.

### 3.4. Histological and Immunohistochemical Findings

The recorded lesion sizes demonstrated a broad range, extending from approximately 0.10 cm to 9.0 cm, with a mean diameter of 3.16 cm. This distribution reflects substantial heterogeneity in lesion dimensions, spanning from very small findings to large multiloculated masses. Of the total 69 reported cases, all three tissue types—endometriosis, endosalpingiosis, and endocervicosis—were identified in 22 cases. In over one-third of the cases, two of the three components were observed concurrently. Among the three tissue types, endometrial-type glands were the most frequently reported component, highlighting its predominant occurrence relative to endosalpingiosis and endocervicosis in the studied cohort.

Although Young and Clement define müllerianosis as requiring the presence of at least two of the three Müllerian-derived tissue types for diagnosis, numerous published reports describe isolated cases of endocervicosis or endosalpingiosis [1]. Batt et al. later refined this broader definition, characterizing a choristoma composed of Müllerian rests (normal endometrium, normal endosalpingeal tissue, and normal endocervical tissue) incorporated singly or in combination into other normal organs during organogenesis. They emphasize that a single-tissue form of müllerianosis can be diagnosed with a high degree of confidence only when three criteria are satisfied: the absence of pelvic endometriosis, no direct communication with the endocervix, endometrium, or endosalpinx, and no prior surgical intervention involving the reproductive organs [39]. In our case, two of the three Müllerian tissue types—endometrial and tubal—were identified, fulfilling the diagnostic criteria proposed by Young and Clement as well as those outlined by Batt et al.

The immunohistochemical profiles were reported for multiple markers, including ER, PR, CK7, CK20, CD10, PAX8, Ki-67, and other ancillary markers (Supplementary Materials). A substantial proportion of cases demonstrated positivity for estrogen receptor and progesterone receptor, particularly in the glandular components, reflecting the hormone-responsive nature of Müllerian-derived tissues. Specifically, ER and PR positivity was observed in the majority of reported cases, often in combination with CD10 positivity in the stromal compartment, confirming the presence of endometrial-type stroma. CK7 expression was consistently positive in the Müllerian epithelium, whereas CK20 was predominantly negative, helping to differentiate these lesions from gastrointestinal or urothelial-derived tissues. PAX8 immunoreactivity was frequently observed in glandular elements, supporting the Müllerian origin of the lesions. Ki-67 proliferation indices were generally low, consistent with the benign or low-proliferative nature of these lesions.

### 3.5. Management and Follow-Up

Most müllerianosis lesions are primarily managed with surgical excision, with complete resection typically leading to durable symptom relief. Transurethral resection is the most common treatment, especially for lesions located in the lumen of the posterior bladder wall. Deeper or infiltrative lesions, including those involving the ureter, often require partial cystectomy, ureteral resection, or combined hysterectomy [4]. Hormonal therapy (GnRH agonists or aromatase inhibitors) may reduce lesion size or control symptoms when surgery is not feasible or incomplete [9,10,40,41]. The majority of patients remain symptom-free after adequate treatment.

Most reported cases support the benign nature of this condition. However, one reported case suggests that endocervicosis, although generally considered a benign lesion, may rarely undergo malignant transformation into adenocarcinoma [42]. Considerably more attention has been directed toward the malignant potential of endosalpingiosis. Chui et al. suggested that endosalpingiosis shows how cancer-related mutations can occur even in cells that appear normal under the microscope. These changes may make such cells more prone to transformation into neoplastic lesions, especially when normal protective mechanisms are lost. They highlighted the role of BRAF and KRAS mutations, which are commonly found in ovarian low-grade serous neoplasms [43]. Moreover, a case of endometrioid adenocarcinoma of the urinary bladder arising in the background of müllerianosis has been reported, underscoring both the rarity of such transformation and the importance of careful morphological assessment and additional analyses in establishing the correct diagnosis [44].

Recurrences have been reported in four cases, which were subsequently treated with repeat TUR or partial cystectomy. However, follow-up in most cases was either short or not reported, with a duration ranging from 3 to 12 months. Our case is unique due to an 11-year follow-up, the longest documented. During this period, the patient experienced three successful pregnancies, resulting in three healthy children. Notably, at the time of hysterectomy, she was found to have developed adenomyosis and endometriosis, raising the question of whether these conditions were etiologically related to prior surgical interventions or whether they share a common pathogenesis with müllerianosis, potentially arising through the metaplasia theory. The temporal relationship between pregnancy and cesarean delivery suggests that hormonal and mechanical factors may have modulated disease expression. The high-progesterone state of three pregnancies may have temporarily suppressed Müllerian lesion activity, whereas repeated cesarean-related injury and repair responses may have promoted inflammation, fibrosis, and tissue remodeling, contributing to aggressive secondary adenomyosis. Thus, müllerianosis, endometriosis, and adenomyosis may reflect both shared Müllerian pathogenesis and subsequent modification by hormonal and surgical factors.

Given the extreme rarity of müllerianosis and the limited number of reported cases, multicenter collaboration and standardized prospective collection of clinical, imaging, histopathological, and follow-up data would be valuable. Such collaborative studies could help better characterize the natural history of the disease, clarify its relationship with other Müllerian lesions, and improve the preoperative differentiation of müllerianosis from more common benign and malignant uterine masses.

## 4. Conclusions

In conclusion, müllerianosis is a rare, benign condition of ectopic Müllerian tissues that can mimic malignancy and present variably depending on location. Diagnosis requires careful imaging, histopathology, and immunohistochemistry, while complete surgical excision remains the mainstay of treatment. Our case is notable for the unusually long follow-up, the uncommon anterior uterine location, and the large size of the lesion, as well as the subsequent development of adenomyosis and endometriosis. These findings support an embryologic or metaplastic origin of müllerianosis and suggest potential links with other uterine pathologies.

## Data Availability

This is a descriptive case report. No research data was published here, so there are no relevant data to share.

## Supplementary Materials

The following supporting information can be downloaded at: https://www.mdpi.com/article/10.3390/jcm15176918/s1, Table S1: Summary of Clinical and Histopathological Findings [1,4,8,9,10,14,15,16,17,18,19,20,21,22,24,25,26,27,28,29,30,31,32,33,34,35,36,40,41,42,44,45,46,47,48,49,50,51,52,53,54,55,56,57,58,59,60,61,62,63,64,65,66,67,68,69,70,71,72,73,74,75].
