# Supplementary material for: Uterine Müllerianosis
Source: J Clin Med. 2026 Sep 7;15(17):6918. doi: 10.3390/jcm15176918 (PMC13566048; doi:10.3390/jcm15176918)
Supplement: Supplementary file 1 [file jcm-15-06918-s001.zip › jcm-4485660-supplementary.pdf]

Table S1. Summary of Clinical and Histopathological Findings.

	First Author, Year	No. of Cases	Patient Age	Previous pelvic operation	Location	Size (cm)	Clinical Presentation	E	S	C	IHC	Treatment	Outcome / Follow-up
**1**	Suzuki R., 2025[28]	1	49	NO	Subcutaneous left buttock	4	Palpable, painless subcutaneous mass for >15 years; discomfort when sitting	-	+	-	PAX8 +, WT-1 +; Alcian blue +	Surgical excision under local anaesthesia	Symptoms resolved
**2**	Quillen K. et al., 2025[4]	1	57	NO	Left distal ureter and parametrium	3	Severe left hydroureteronephrosis; mass suspected to be malignant	-	+	+	ND	TAH with partial ureteral resection and re-implantation	postoperative course described as uneventful
**3**	Aydın Ö. et al., 2025[54]	1	53	ND	Posterior wall of bladder	ND	Left flank pain; incidental bladder mass on USG	**ND**	**ND**	**ND**	ND	ND	the lesion recurred in two further cystoscopies within 2 years
**4**	Ding D. et al., 2025[29]	1	11	NO	Appendix	ND	Symptoms suggestive of appendicitis	-	+	-	ND	Appendectomy	ND
**5**	Canete-Portillo S. et al., 2025[71]	1	66	SC, TAH+BSO [endometriosis]	distal ureter	ND	Hydroureteronep-hrosis	-	+	+	ER+ , PAX8+	Surgical resection of ureteral lesion	ND
**6**	Sánchez Godoy L. et al, 2024[45]	1	49	ND	Bladder [posterior wall]	0.5	Recurrent UTIs, urinary discomfort, abdominal pain	**ND**	**ND**	**ND**	ND	ND	ND
**7**	Panackel C. et al., 2024[30]	1	41	ND	Liver and lung	ND	Mimicked metastatic biliary cystadenocarcinoma	**ND**	**ND**	**ND**	ND	Surgical resection of lesions	ND
**8**	Liu Z, 2024[26]	1	36	ND	Anal canal [mass between anal canal and vagina]	3.5x3.5	2-year history of difficult defecation and intermittent bloody stool; protruding anal mass	+	-	+	PAX8+	Surgical excision of mass	6 mo follow-up

**9**	Wegrzyn G.H. et al., 2024[46]	1	65	3 SC	posterior bladder wall /trigonal ridge	2-3	Incidental bladder mass; asymptomatic	**ND**	**ND**	**ND**	CK7 +; PAX8 +; CK20 -; GATA3+/-	TURB	ND
**10**	Ghosh S. et al., 2021[18]	1	31	NO	posterior wall of the urinary bladder	2-4	pain during micturition and increased urinary frequency during menstrual cycles	**+**	**+**	**-**	CD10 focal +, ER+, PR+	TURB	12 mo follow-up
**11**	Bocchialini T. et al., 2021[55]	1	61	appendicectomy and SC	left ureteric orifice	1.8x1.2	hypogastric pain and dysuria	**-**	**+**	**-**	ND	TURB	12 mo follow-up
**12**	Fakhralddin S.S. et al., 2021[47]	1	38	3 SC	posterior wall of the urinary bladder	1.3x1.0	Hematuria, dysuria, pain	**-**	**+**	**+**	ND	TURB	3 mo follow-up
**13**	Almatrafi M.H. et al., 2020[19]	1	32	ND	posterior wall of the urinary bladder	3	Periodic hematuria, dysuria	**+**	**+**	**+**	ND	TURB	ND
**14**	Mahajan A.D., et al., 2020[56]	1	25	SC	antero-superior part of the urinary bladder, extending anteriorly in the prevesical space and into the anterior abdominal wall	5x2.9	dysuria and lower abdominal pain	**+**	**+**	**-**	ND	excision of the mass with partial cystectomy	Symptoms resolved
**15**	Sancheti S. et al. 2020[20]	1	37	NO	left posterior wall of bladder	3.3x2.1	dysuria, burning micturition 10 days before menstruation	**+**	**-**	**+**	ER+; CD10+	biopsy of lesion	ND
**16**	Rajaian S. et al., 2020[57]	1	45	NO	Urinary bladder [dome & left posterolateral wall] + unilateral left renal agenesis	4x4	dysuria and lower abdominal discomfort	**+**	**-**	**+**	CA125 +; ER/PR+; GATA3 - [positive in urothelium]	TURB	12 mo follow-up
**17**	Subbaiah M. et al., 2020[31]	1	58	NO	uterine surface	0.1–0.2	postmenopausal bleeding	**-**	**+**	**-**	ND	TAH+BSO	ND
**18**	Jhang S., Chen M., Chen L.-C., 2020[48]	1	43	NO	Urinary bladder [posterior wall] and umbilicus	2.4	Intermittent umbilical bleeding; bladder lesion incidentally found	**+**	**+**	**-**	ER +; CD10 +	TURB	6-month follow-up

**19**	Salada R.B., et al., 2019[21]	1	33	SC	posterior wall of the urinary bladder	1.3x1.0	Painless hematuria associated with menstruation	+	+	+	ER+; CD10 +	TURB	ND
**20**	Yang M, et al. 2019[35]	1	31	3 vacuum curettage	Uterus [posterior wall]	7.7x7.6	abdominal mass, AUB, pelvic pain	-	+	-	ND	laparoscopic resection	ND
**21**	Gilbert N., et al., 2018[49]	1	40	TAH without adnexectomy+ Iatrogenic bladder injury; 3 SC	posterior wall of the urinary bladder	2	Painful gross haematuria	-	+	-	ND	TURB	3 mo follow-up
**22**	Amir R.A.R., et al., 2018[22]	1	40	NO	posteromedial bladder wall near ureteric orifice	4 × 3.5	Urgency/urge incontinence during menstruation; gross hematuria	+	+	+	ER+, PR+, CD10 +	lesion resection	ND
**23**	Quirante F.P., et al., 2017[32]	1	35	laparoscopic cholecystectomy, laparoscopic supracervical hysterectomy and bilateral salpingectomy	Appendix	ND	right iliac fossa pain; initially suspected appendicitis.	+	+	+	ER+, PR+	ileocecectomy	ND
**24**	Iida Y., et al., 2017[70]	1	44	NO	obturator lymph nodes	1.5	swelling in the lower abdomen	+	+	-	ND	TAH+BSO, omentectomy, lymphaden-ectomy	ND
**25**	Santos-Lopes S., et al., 2017[58]	1	35	SC; subtotal hysterectomy & oophorectomy.	Urinary bladder [right lateral wall / dome]	2.1x1.6	asymptomatic	-	-	+	ER+, PR+	TURB	12 mo follow-up
**26**	Lee S.H., et al., 2017[59]	1	50	NO	Cervix	7.5x6.2	Abnormal vaginal bleeding; cervical mass detected on examination	-	-	+	ER/PR+, CK 7+, CEA-, MUC6-, P53-, P16-; low expression Ki 67	Surgical excision of cervical lesion	ND
**27**	Patel A., et al, 2017[60]	1	59	NO	Urinary bladder [dome]	2.8x3	Painful haematuria, urgency, frequency, incontinence	-	+	+	PAX8+, ER+, PR+	TURB	yes [part. cystectomy]
**28**	Rosell Malchirant M.I., et al., 2016[27]	1	34	NO	Urinary bladder [left lateral wall and dome]	4.5x3	4-month history of worsening dysmenorrhea, dysuria, and intermittent hematuria [cyclical pattern]	+	+	+	ND	TURB; recurrence → part. cystectomy + TAH +resection of endometriotic implants	Symptom-free for 1 year; later new left ovarian cyst under evaluation

**29**	Val-Bernal J-F., et al., 2016[72]	1	59	hysterectomy	vaginal cuff	1.5	Incidental finding during surgery for rectal adenocarcinoma	+	+	+	CK7+, CK8/18+, H-MW CK+, Erα+, AR+; stroma SMA+, h-caldesmon+, CD10+	Surgical excision of vaginal cuff mass	3 mo follow-up
**30**	Nakaguro M., et al., 2016[42]	1	58	total hysterectomy and left salpingo-oophorectomy	posterior wall of the urinary bladder	4	Asymptomatic hematuria	-	-	+	CK7+,AE1/AE3+, CAM5.2+, HBME1+, CA19-9+; carcinoma: loss of ER expression and higher Ki-67 index	Total cystectomy	ND
**31**	Stanimir M., et al., 2016[69]	1	64	hysterectomy, SC and appendectomy	left bladder wall around left ureteric meatus	1.6	Left lower abdominal pain, recurrent UTIs, urinary incontinence, hematuria	+	+	+	ND	TURBT → followed by partial cystectomy + left ureter re-implantation	two years later hernia surgery
**32**	Morales-Roselló J., et al., 2016[27]	1	30	NO	the uterine fundus	ND	NO	-	+	-	PAX8+, CK7+, ER+, PR+; CD10-, calretinin-, CK20-; Ki67 < 1%.	Surgical excision after delivery	ND
**33**	Rajarubendra N., et al., 2015[50]	1	30	ectopic pregnancy	posterior wall of the urinary bladder	3.9x3.7	right iliac fossa pain	-	+	+	ND	partial cystectomy	recurrence after 2mo
**34**	Del Carmen S., et al., 2015[68]	1	62	ND	retroperitoneal tumor adherent to sigmoid serosa	1.3x8	Left inguinal/pelvic pain for 2 months	+	+	+	CK7+, ER+, PR+; CK20-, CEA-	TAH+BSO resection of pelvic mass	4 mo follow-up
**35**	Casasayas-Carles P., et al., 2014[61]	3	31–34	case 1: SC	Bladder	4.5x3; 2; 2.5	Case 1 & 3: hematuria, pelvic pain; Case 2: recto-vaginal endometriosis surgery	+	+	+	ND	TURBT for superficial lesions; partial cystectomy in infiltrative cases; TAH excision of endometriotic implants in selected patients	Case 1: asymptomatic at 6 mo post-op; Case 2: asymptomatic, no reintervention; Case 3: asymptomatic after TURBT
**36**	Hemalatha A.L., et al., 2014[33]	1	40	ND	Uterine parametrium	7.5 x 5	Pelvic pain	-	+	-	ND	Hysterectomy	ND
**37**	Jayalakshmy P.S., et al., 2014[36]	1	48	NO	Uterus [fundus, serosal surface]	9x9	Lower abdominal mass/swelling	+	+	-	CK +; calretinin - low Ki-67 index	TAH+BSO	postoperative course uneventful
**38**	Maeda K, et al., 2014[24]	2	39; 37	Case 1: none; Case 2: previous ovarian cyst resection and rectal endometriosis surgery	Urinary bladder, posterior wall [both cases]	2.5; 2.0	Case 1: menstrual dysuria; Case 2: incidental finding [asymptomatic]	+	+	+	ER+, PR+ , CD10+ , CK7+, CK20−, low Ki-67	TURB	Symptom resolution; no recurrence reported during short follow-up

**39**	Scott W.W., et al., 2014[9]	1	29	Yes – spinal surgery in childhood [for presumed ependymoma]	Spinal cord – conus medullaris	ND	Progressive bilateral lower extremity weakness, urinary incontinence; cyclical neurological symptoms	+	-	-	CAM 5.2+, PAX8+, ER+, PR+; SMA+; GFAP in glia; negative for CDX2, TTF1, CEA, inhibin	fenestration, cyst drainage, cysto-subarachnoid shunt lesion resection, postoperative leuprolide for 6 months	Modest recovery of motor function; persistent neurogenic bowel/bladder dysfunction
**40**	Rajakumar C., et al., 2014[51]	1	32	NO	Posterior wall of bladder	3	chronic pelvic pain, dysmenorrhea	-	-	+	CK7+, CK20-	TURB, LPSC Partial Cystectomy; Robotically Assisted Bladder Repair	12 mo follow-up
**41**	Oida et al., 2013[67]	1	35	myomectomy	right posterior wall	2	NO	-	+	+	ER+	TURB, recurrence - partial cystectomy	recurrence after 8mo, then 32 mo follow-up
**42**	Mishima et al., 2013[66]	1	39	NO	Posterior wall of bladder	2,5	difficulty in defecation	+	+	-	ER+, PR+	TURB, then partial cystectomy	10 mo follow-up
**43**	McSorley S., et al., 2013[52]	1	61	2 SC	Posterior wall of bladder	2.4x2.0	dysuria, urinary frequency, microscopic hematuria	+	+	-	Epithelium CK7+, ER+, PR+, CD10+	TURB	ND
**44**	Guan H., et al., 2012[65]	1	28	NO	left lateral wall of the bladder	3x2.2	Hematuria; bladder mass;	+	-	+	ND	Surgical resection	ND
**45**	Ndokera R., et al., 2012[73]	1	50	laparoscopic subtotal hysterectomy	left vesico-ureteric junction	2	Acute renal colic [left-sided]	+	+	-	CD10 +	TURB	3-mo follow-up
**46**	Kudva R., et al., 2012[41]	1	32	NO	right lateral wall of bladder	4	dysuria and burning micturition	+	+	+	ND	TURB; GnRH analogue	24-mo follow-up; lesion was persistent which was smaller in size
**47**	Ogah K., et al., 2012[25]	1	45	SC	Posterior wall of bladder	2.3x2.1	chronic pelvic pain, dysmenorrhoea, irritative lower urinary tract symptoms and cyclical haematuria	+	+	+	ND	TAH, partial cystectomy	3-mo follow-up

**48**	Olivia Vella, et al., 2011[40]	2	70, 43	Case 1: subtotal hysterectomy + BSO; Case 2: multiple laparotomies for endometriosis	Urinary bladder [trigone for Case 1; inferior bladder mucosa for Case 2]	2	Case 1: asymptomatic vaginal bleeding; Case 2: dysuria, urinary frequency, pelvic pain, long-standing endometriosis	+	+	+	CK7 +; CD10 +, ER/PR +;	Case 1: medical therapy with Arimidex; Case 2: cystoscopic excision	Case 1: 18-month follow-up, lesions reduced, vaginal bleeding ceased; Case 2: 4-month follow-up, symptom-free
**49**	Batt R.E.,et al., 2010[75]	1	41	NO	Posterior mediastinal paravertebral region [T6]	2.1x1.5	Left-sided mid-thoracic pain associated with moderate rotary scoliosis	-	+	-	AE1/3+, CK7+, EMA+, ER+, PR+; CK20-, calretinin-	Surgical excision via left thoracotomy	no recurrence reported during short follow-up
**50**	Maniar K.P., et al., 2010[53]	1	54	Prior pelvic surgery with traumatic bladder injury [~30 years prior]	Posterior wall of bladder	2.5	Dysuria, chronic lower urinary tract symptoms worsening over 6 months	-	+	-	CK7+, KER903+, ER+, PR+, patchy p63+; CK20-, CD10-, PSA-, P504S-, prostatic acid phosphatase-	TURB	ND
**51**	Abern M.R., et al., 2009[64]	1	84	hysterectomy	Left proximal ureter	ND	Vague abdominal pain localized to epigastrium; no hematuria	-	+	-	ND	Endoscopic biopsy with fulguration of base of lesion	36-mo follow-up
**52**	Cruz Guerra et al., 2009[62]	1	30	History of miscarriage	Urinary bladder [posterolateral wall]	2.4	Voiding disturbances coinciding with menstruation, mild hematuria	+	-	+	ND	TURB	12 mo follow-up
**53**	Li W-M., et al., 2007[63]	1	49	2 SC	Ureter	4x1	flank pain, dysuria, or hematuria or fever and chills	+	+	+	ND	Segmental ureteral resection	3-mo follow-up
**54**	Barresi V., et al., 2006[10]	1	42	ND	Intradural spinal cord [L2-L3]	1.9	Catamenial lumbago and sciatica [3 years], left Achilles tendon areflexia	+	+	+	ER+, PR+, vimentin+, low molecular weight cytokeratins+, CA 19.9+	Surgical excision [L2-L3 laminectomy], followed by hormonal therapy	Post-op: complete remission of symptoms; 6-month MRI normal
**55**	Koren J., et al., 2006[17]	1	41	NO	Posterior wall of bladder	1.5	Dysuria, chronic pelvic pain, hematuria [5 years]	+	+	+	ER+, PR+ ,CK7+, AE1/AE3+; CD10+; vimentin +	TURB	ND
**56**	Garavan F, et al., 2004[44]	1	53	ND	Posterior wall of bladder	1	intermittent painful gross haematuria	+	+	+	CD10 +/-	TURBT → TAH + partial cystectomy	ND
**57**	Islam S., et al., 2003[15]	1	37	2 SC; left salpingooophore-	Posterior wall of bladder	2x3	vaginal discharge and right iliac fossa pain	+	+	+	ND	TURB	ND

				ctomy for a torted ovarian cyst									
**58**	Lim S., et al., 2003[34]	1	37	NO	left mesosalpinx	2.2	intermittent left–lower-quadrant pain, dysmenorrhea, and leukorrhea.	**+**	**+**	**+**	ND	TAH+BSO	ND
**59**	Sinkre P., et al., 2002[74]	1	48	ND	Right inguinal lymph nodes	2.5	symptomatic uterine leiomyomas; 2.5-cm, right, firm, groin mass	**-**	**+**	**+**	CEA+, ER+	TAH+BSO	24-MO follow-up
**60**	Jiménez-Heffernan J.A., et al., 2000[14]	1	38	Laparoscopic hysterectomy	extrinsic bladder lesion	2.5x 2	pelvic discomfort and dysuria	**+**	**+**	**-**	ND	lesion excision	5-mo follow-up
**61**	Nogales F.F., et al., 1999[16]	1	39	NO	Right ureter	5.3x 1.5	acute right ureteral colic	**+**	**+**	**+**	ER+, PR+, OC125+, CA19-9+, vimentin+, CAM 5.2 cytokeratins+	Segmental ureteral resection with ureterovesical reimplantation	10-year history before resection
**62**	Donné C., et al., 1998[8]	1	ND	ND	Posterior wall of bladder	4.5x4.0	Lower urinary tract symptoms [dysuria/hematuria]	**+**	**+**	**+**	ER+, PR+	TURB	ND
**63**	Young R.H., Clement P.B., 1996[1]	3	37, 44, 46	ND	Posterior wall of bladder	4	Masses up to 4 cm; lower urinary tract symptoms	**+**	**+**	**+**	ND	TURB	ND

ND = Not described / Not documented; **SC** = Cesarean section; **TAH** = Total abdominal hysterectomy; (**B)SO** = (Bilateral) salpingo-oophorectomy; **USG** = Ultrasonography; **AUB** = Abnormal uterine bleeding; **UTI** = Urinary tract infection; **TURB** = Transurethral resection of bladder tumor; **LPSC** = Laparoscopic; **E** = Endometrial tissue; **S** = Endosalpingial tissue; **C** = Endocervical tissue; **IHC** = Immunohistochemistry; **ER** = Estrogen receptor; **PR** = Progesterone receptor; **PAX8** = Paired box gene 8; **WT-1** = Wilms' tumor 1 protein; **CK7** = Cytokeratin 7; **CK20** = Cytokeratin 20; **GATA3** = GATA-binding protein 3; **CD10** = Cluster of Differentiation 10; **CAM5.2** = Low molecular weight cytokeratin; **SMA** = Smooth muscle actin; **GFAP** = Glial fibrillary acidic protein; **CEA** = Carcinoembryonic antigen; **MUC6** = Mucin 6; **Ki-67** = Proliferation marker; **AR** = Androgen receptor; **CA125** = Cancer antigen 125; **CA19-9** = Cancer antigen 19-9; **EMA** = Epithelial membrane antigen; **P504S / AMACR** = Alpha-methylacyl-CoA racemase.
